# Influence of Physical Self-Concept and Motivational Processes on Moderate-to-Vigorous Physical Activity of Adolescents

**DOI:** 10.3389/fpsyg.2021.685612

**Published:** 2021-08-12

**Authors:** Juan L. Núñez, Jaime Leon, Alfonso Valero-Valenzuela, Luis Conte, Juan A. Moreno-Murcia, Elisa Huéscar

**Affiliations:** ^1^Department of Psychology, Sociology and Social Work, University of Las Palmas de Gran Canaria, Las Palmas de Gran Canaria, Spain; ^2^Department of Education, University of Las Palmas de Gran Canaria, Las Palmas de Gran Canaria, Spain; ^3^Department of Physical Activity and Sports, University of Murcia, Murcia, Spain; ^4^Sports Research Center, Miguel Hernández University of Elche, Elche, Spain; ^5^Department of Health Psychology, Miguel Hernández University of Elche, Elche, Spain

**Keywords:** physical self-concept, need satisfaction, autonomous motivation, moderate-to-vigorous physical activity, adolescence

## Abstract

There is a growing concern about the increasing decline in physical activity among adolescents. In the search for variables that may be related to physical activity, this study examined the influence of physical self-concept on objectively measured moderate-to-vigorous physical activity (MVPA) of adolescents through the mediation of the needs satisfaction and two types of autonomous motivation, for academics and for physical education. Data were collected from 618 students (301 boys and 317 girls) aged 10–14 years from 24 secondary schools in Spain. The path analysis results showed that physical self-concept positively predicted needs satisfaction and this, in turn, was positively and significantly related to the two types of autonomous motivation. Finally, only the autonomous motivation for physical education significantly and positively predicted the adolescents’ MVPA. Our findings showed that there was no evidence of an indirect effect of physical self-concept on MVPA. The results are discussed along the lines of the self-determination theory, through the analysis of the role of physical self-concept in increasing adolescents’ physical activity.

## Introduction

According to the WHO, more than 80% of in-school adolescents aged 11–17 years worldwide do not meet the recommendations of at least 1 h of physical activity per day, thereby compromising their present and future health. If this trend continues, the global target of a 15% relative reduction in insufficient physical activity and a global prevalence of less than 70% will be missed by 2030 (Guthold et al., 2020). This reveals the need for urgent action to increase the level of physical activity of adolescents. The scientific literature has shown evidence that moderate-to-vigorous physical activity (MVPA), compared to lower intensity physical activity, has favorable effects on the health of adolescents (Piercy et al., 2018; García-Hermoso et al., 2021), with numerous benefits, such as lower fat gain, improved cardiorespiratory fitness, and better mental health (Kalajas-Tilga et al., 2020). In spite of this, a decline in the level of physical activity has been observed as the age of the adolescents increases (Dumith et al., 2011). An important aim of research on adolescent health and quality of life is to identify the psychological and social aspects related to participation in actions that require physical activity (Ensrud-Skraastad and Haga, 2020). This study aims to contribute to this objective by analyzing the influence of personality variables, such as physical self-concept, and motivational processes, such as satisfaction of basic psychological needs and autonomous motivation, for both academic and physical activity, on adolescents’ MVPA.

## Physical Self-Concept

Self-concept has occupied an essential place in the explanation of human behavior in different contexts. It is related to phenomena that mirror a mentally healthy life and effective adaptation, which enhances psychological wellbeing. It also protects an individual against stressors that can cause physical and psychological pathologies (Garaigordobil et al., 2005). Although some authors have described the idea that self-concept is structured into a single dimension (Coopersmith, 1967), the strongest empirical support has been provided to the description of self-concept as a multidimensional and hierarchical structure (Byrne, 1984). Within this approach, Shavelson et al. (1976) established a hierarchical and multidimensional model in which self-concept is considered as an individual’s perception of him/herself, based on his/her experiences or relationships with others and attributions of his/her own behavior. Within this model, self-concept is divided it into academic self-concept and non-academic self-concept, the latter being subdivided into emotional, social, and physical components. Fox and Corbin (1989) stated that this notion of multidimensionality implicitly supports the recognition of an independent physical self-concept and establishes four specific sub-domains: sports competence, attractive body, physical strength, and physical condition. Self-concept in specific domains influences behavioral choice and the subsequent persistence in that domain (Harter, 1999).

Adolescence is a crucial period in the development of self-concept and is considered a key element for physical education teachers (Cheon et al., 2019), because a positive physical self-concept is associated with increased physical activity (Marsh et al., 2006). A positive physical self-concept has important consequences for young people’s physical activity behavior; in particular, self-perceived physical condition and sports competence have been strongly associated with physical activity (Crocker et al., 2006). Babic et al. (2014) concluded, in a systematic review, that children and adolescents with high levels of physical self-concept were more likely to participate in physical activity. Furthermore, Taylor et al. (2014) found that autonomous motives for physical education (i.e., intrinsic and identified motives) were positively associated with physical self-concept. This relationship has also been observed in previous cross-sectional studies (Standage et al., 2012).

## Autonomous Motivation

The organismic integration theory is a sub-theory from the self-determination theory (SDT) that specifies that motivation is a multidimensional construct that varies along a continuum, depending on the self-determination or internalization of the activity. Thus, Deci and Ryan (1985) proposed different types of motivation, which, ordered from the highest to the lowest level of internalization, are as follows: intrinsic motivation, it involves engagement in an activity for the pleasure and enjoyment it produces, and there is an inherent interest and the activity is voluntarily carried out; and four sub-types of extrinsic motivation: (i) integrated regulation, which implies congruence of the activity with personal values and interests; (ii) identified motivation, in which the person identifies with the value of the activity; (iii) introjected motivation, which involves internal coercion or pressure to do something, the person acts to avoid anxiety, shame, or guilt; and (iv) external regulation, the typical non-autonomous motivation, whose behavior is controlled by external rewards and punishments. Finally, amotivation is the absolute lack of intention and motivation, both intrinsic and extrinsic; it represents the lowest degree of self-determination. Due to the quality of being highly volitional, autonomous motivation is represented by intrinsic motivation, and integrated and identified regulation (Ryan and Deci, 2020). In the academic context, the SDT postulates, on the one hand, that more autonomous forms of motivation lead to more positive consequences (e.g., improved student engagement, learning, and wellbeing) and, on the other hand, that the satisfaction of basic psychological needs facilitates such motivation (Ryan and Deci, 2020). These needs are autonomy, competence, and relatedness. Autonomy refers to having initiative; an individual is the owner of his/her own actions. Competence pertains to the feeling of mastery; the subject feels that he/she can grow and improve. Finally, relatedness is concerned with a sense of belonging; the subject feels connected to others. SDT argues that the satisfaction of needs improves intrinsic motivation and internalization, and the frustration of any of these three basic needs significantly undermines autonomous motivation (Ryan and Deci, 2020). Personal autonomous motivation has been positively associated to objectively measured MVPA levels in adolescents (Aelterman et al., 2012). Along the same line, Sebire et al. (2013) have demonstrated that intrinsic motivation was positively associated to objectively measured MVPA in children, and more recently, Kalajas-Tilga et al. (2020) showed that intrinsic motivation was positively and significantly related to objectively measured MVPA of adolescents.

## The Present Study

The present work aims to expand the knowledge in this area of study by integrating personal and motivational variables into a unified model, to analyze their influence on an objective measure of MVPA, and to improve the health and quality of life of adolescents. The inclusion of physical self-concept in the model, as a predictor or antecedent variable of needs satisfaction, and the incorporation of two types of autonomous motivation (i.e., academic and physical education), which have an influence on objectively measured MVPA, are innovative elements of this research study. To date, we are not aware of works that consider two types of autonomous motivation within the same model. In this sense, it is of interest to test not only the influence of the motives for participation in physical education classes on MVPA, but also the effects of the motives for going to high school; in other words, the possible influence of a more general context is beyond the physical education class.

**Figure 1:**
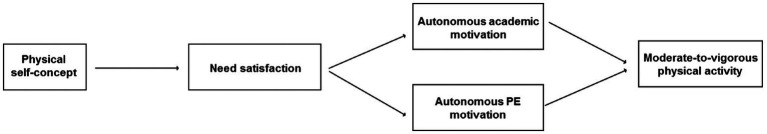
Hypothesized model.

The hypothesized model (Figure 1) states that physical self-concept influences the needs satisfaction, and this, in turn, influences the academic autonomous motivation and autonomous motivation for physical education. Finally, these two types of autonomous motivation have an influence on adolescents’ MVPA.

According to this model, we hypothesize that as:

*H1:* The physical self-concept positively predicts the needs satisfaction.

*H2:* The needs satisfaction positively predicts autonomous motivation, for academics and physical education.

*H3:* The autonomous academic motivation and the autonomous motivation for physical education positively predict adolescents’ MVPA.

*H4:* The physical self-concept perceived by adolescents is positively and indirectly related to adolescents’ MVPA through needs satisfaction and autonomous motivation (for academics and physical education).

## Materials and Methods

### Participants

After eliminating the cases that did not meet the minimum criteria requirements explained in the procedure (see below), the final sample consisted of 618 students (301 boys and 317 girls) aged 10–14 years (*M* = 11.62, *SD* = 0.94) from 24 public secondary schools in various Spanish municipalities. In Spain, all secondary schools have a similar physical education curriculum, and the same number of hours is dedicated to physical education throughout the country.

### Measures

#### Physical Self-Concept

The Spanish version (Moreno and Cervelló, 2005) of the Physical Self-Perception Profile (Fox and Corbin, 1989) was used. The instrument is composed of 30 items and five factors: sports competence (e.g., I try hard to be the best in class), attractive body (e.g., I have a good figure), physical condition (e.g., I am physically strong), physical strength (e.g., My muscles are as strong as those of most people of the same sex), and self-confidence (e.g., I feel safe learning new skills). The items are preceded by the sentence “When I engage in physical activity…” The response options range from 1 (*strongly disagree*) to 4 (*strongly agree*). The Cronbach’s alphas obtained were 0.76 for perceived competence, 0.81 for attractive body, 0.87 for physical fitness, 0.68 for physical strength, and 0.77 for self-confidence.

#### Needs Satisfaction

The Spanish version (Moreno et al., 2008) of the Basic Psychological Needs in Exercise Scale (Vlachopoulos and Michailidou, 2006), adapted to physical education, was used. The scale consists of 12 items that answer the following statement: “In classes…” The items are grouped into three factors (four items per factor) which assess as: autonomy (e.g., “I feel that the way I exercise is the way I want to”), competence (e.g., “I feel that exercise is an activity in which I do very well”), and relatedness (e.g., “I feel that I have an excellent communication with the people I exercise with”). The items were scored with a Likert-type scale, ranging from 1 (strongly disagree) to 7 (strongly agree). The reliability values were 0.73 for autonomy, 0.77 for competence, and 0.81 for relatedness.

#### Autonomous Motivation for Physical Education

Two subscales of the Spanish version (Moreno et al., 2009) of the Perceived Locus of Causality Scale (Goudas et al., 1994) were used. Each of the subscales consisted of four items: identified regulation (e.g., “because I want to learn sport skills”) and intrinsic motivation (e.g., “because physical education is fun”). This scale was headed by the statement “I participate in physical education classes…” The items were scored with a Likert-type scale, ranging from 1 (*strongly disagree*) to 7 (*strongly agree*). Cronbach’s alpha values were 0.74 for intrinsic motivation and 0.73 for identified regulation.

#### Academic Autonomous Motivation

From the Spanish version (Núñez et al., 2005) of the Academic Motivational Scale (Vallerand et al., 1989), the following subscales were used as: identified regulation (e.g., Because this will help me make a better choice regarding my career orientation), intrinsic motivation to know (e.g., Because I experience pleasure and satisfaction while learning new things), intrinsic motivation to accomplish (e.g., For the pleasure I experience while surpassing myself in my studies), and intrinsic motivation to stimulating experiences (e.g., For the pleasure that I experience when I read interesting authors). Each of the subscales consists of four items and answers the question “Why do you go to secondary school?” and was scored with a 7-point Likert-type scale, ranging from 1 (*does not correspond at all*) to 7 (*corresponds exactly*). The internal consistencies were 0.78, 81, 0.78, and 0.77, respectively.

#### Moderate-to-Vigorous Physical Activity

The students’ MVPA was measured using an accelerometer. For this purpose, the ActiGraph GT3X-plus accelerometer model (4.6 cm × 3.3 cm × 1.5 cm) was used. This accelerometer is a valid and reliable tool for quantifying the adolescents’ physical activity (Reilly et al., 2006). It is a solid triaxial accelerometer that measures the amount and frequency of human activity in the three orthogonal axes: vertical (y), horizontal left and right (x), and horizontal forward and backward (z; Baptista et al., 2012). The accelerometer recorded the different levels of intensity of the exercise performed by the students in a magnitude ranging from −6G to +6G. The raw data recorded was processed through a digital filter in the program, which limited the accelerometer with a response between 0.25 and 2.5 Hz. According to the recommendations from Evenson et al. (2008), to determine the intensity of physical activity performed by the participants, the accelerometers were programmed with the following cutoff points: < 100 for sedentary activity; from 100 to 2,295 (light); from 2,296 to 4,012 (moderate); and > 4,013 (vigorous). Thus, the minutes of sedentary, light, moderate, and vigorous activity of the participating students were obtained. Evenson’s cutoff points have been used in adolescent samples similar to the present study (Aibar et al., 2013) and are highly recommended, as they provide an optimal estimate of physical activity in children and adolescents aged 5–15 years (Robusto and Trost, 2012).

### Procedure

We contacted the secondary school management team to ask for their collaboration in this study. The students were asked for written authorization from their parents, because they were minors. The scales were administered online in a classroom at their secondary school. These sessions were programmed in collaboration with the physical education teacher and the secondary school’s management team. Prior to the online administration of the scales, the student participants were informed about the objectives of the research, the number of scales, and how to answer the items. In addition, they were informed about the anonymity of their responses and were encouraged to answer honestly. The scales took approximately 20 min to complete.

Before the delivery of the ActiGraphs, two researchers verbally explained the procedure of use of the accelerometers to the participants in an informative meeting. Each participant received an accelerometer to wear for the following 7 days. Similar to previous studies with adolescents (Aibar et al., 2013), and according to the guidelines suggested by Trost et al. (2005), the participants were instructed to start wearing the device on the right hip at the level of the iliac crest, as soon as possible, and to only take it off to sleep, shower, or when participating in water activities. They were to keep a daily record of all these activities, as well as to record if they had forgotten any activity. Following the procedure established by previous studies regarding the duration of the accelerometer recording (Ferrari et al., 2015; Gába et al., 2017), the participants had to have a minimum of 4 days recorded and at least 600 min recorded each day. In addition, it was indicated that any movement lasting less than 2 min would not be considered. Participants who did not reach the minimum required in the established criteria were eliminated from the final sample. The present study was approved by the Ethical Committee of the Miguel Hernandez University of Elche.

### Data Analysis

All data analyses were performed using MPLUS 8.5. First, we computed the mean and standard deviations. To gather evidence of the univariate and multivariate distribution, we computed the skewness and kurtosis of each variable and performed a Mardia test for the multivariate distribution. To explore the nesting of the data, we estimated the intraclass correlation coefficient, and to explore the relationships between variables, we estimated bivariate correlations. Next, to test the hypothesized model, we performed a path analysis. As shown in the results section, the data were non-independent and slightly departed from normality. For the former issue, we corrected the standard errors and the fit indexes by using a sandwich estimator (Muthén and Muthén, 2017), and for the latter, we used the Maximum Likelihood estimator with the Satorra-Bentler correction (Satorra and Bentler, 1994). To estimate the standard errors of the indirect effects, we used the bootstrap method with 1,000 replications.

## Results

### Descriptive Analyses

Means, standard deviations, skew, kurtosis, intraclass correlation coefficient, and bivariate correlations are shown in Table 1. The result of the Mardia test was 32.61 (*p* < 0.001), which indicated non-normality.

**Table 1:** Means, standard deviations, and correlations among variables.

S. No.		*M*	*SD*	ICC	Skew	Kurtosis	1	2	3	4
1.	Physical self-concept	3.29	0.58	0.11	1.15	1.75	–			
2.	Autonomous motivation for physical education	6.36	0.94	0.04	0.87	18.75	0.32^**^	–		
3.	Academic autonomous motivation	6.04	0.87	0.10	−1.00	0.40	0.37^**^	0.37^**^	–	
4.	MVPA	2.01	1.02	0.06	−0.81	0.20	0.05	0.15^*^	0.03	–

* *p* < 0.05;

** *p* < 0.001.

*ICC, Intraclass correlation coefficient*.

### Path Analysis

**Figure 2:**
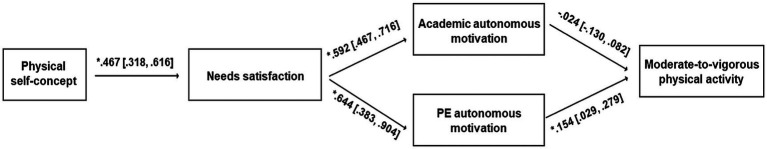
Structural equation model. ^*^The parameters are significant at *p* < 0.05 and standardized.

Figure 2 shows the relationship between the studied variables. For the effect of physical self-concept on needs satisfaction was *β* = 0.467 (0.318, 0.616). The explained variance of needs satisfaction was 0.782 (0.643, 0.921). The effect of the latter on academic autonomous motivation was *β* = 0.592 (0.467, 0.716), with an explained variance of 0.650 (0.502, 0.797), and on autonomous motivation for physical education was *β* = 0.644 (0.383, 0.904) with an explained variance of 0.586 (0.251, 0.921). The effect of academic autonomous motivation on MVPA was *β* = −0.024 (−0.130, 0.082), and for the effect of autonomous motivation for physical education on MVPA was *β* = 0.154 (0.029, 0.279). The explained variance of MVPA was 0.978 (0.943, 1.014).

#### Indirect Effects

For the indirect effect of needs satisfaction on the relationship between physical self-concept and academic autonomous motivation was *β* = 0.276 (0.174, 0.382), while on the relationship between physical self-concept and autonomous motivation for physical education was *β* = 0.301 (0.145, 0.479). The indirect effect of academic autonomous motivation on the relationship between needs satisfaction and MVPA was *β* = 0.099 (0.002, 0.215); regarding, autonomous motivation for physical education in the same relationship was *β* = −0.014 (−0.090, 0.046). Finally, the indirect effect value of needs satisfaction and academic autonomous motivation on the relationship between physical self-concept and MVPA was *β* = 0.046 (0.001, 0.155), and for needs satisfaction and autonomous motivation for physical education in the same relation was *β* = −0.007 (−0.047, 0.026).

## Discussion

The positive effects of MVPA practice are innumerable; however, the level of physical activity decreases considerably during adolescence. To contribute to the improvement of these levels of activity, the main objective of this research was to examine the influence of the physical self-concept on adolescents’ MVPA through motivational processes (needs satisfaction and two types of autonomous motivation).

The results partially confirm the hypotheses posited in the personality variables and motivational processes hypothesized model. Specifically, the (*H1*) “physical self-concept positively predicts needs satisfaction” was confirmed. Thus, the results indicated that the physical self-concept had a positive and significant effect on the basic psychological needs satisfaction (i.e., autonomy, competence, and relatedness). In addition, the variance explained was high. In this case, physical self-concept showed a determining role for motivational processes. Thus, a student who has a positive self-perception on the specific domain of physical activity will satisfy his/her basic psychological needs in physical education class. This result confirms the positive relationships between the three needs and the physical self-concept established by Fraguela-Vale et al. (2020). Along the same line, Cheon et al. (2019) concluded in a longitudinal study that there was a relationship between needs satisfaction and increased physical self-concept, although in this case, physical self-concept was a consequence of basic psychological needs.

According to (*H2*) “the needs satisfaction positively predicts autonomous motivation, for academic and for physical education,” the results of the current study showed that needs satisfaction was positively and significantly associated to the two types of autonomous motivation, academic, and physical education. In both cases, the explained variances were high. This result is in line with the SDT postulates (Ryan and Deci, 2020) and coincides with several previous works within the specific field of physical education (Ntoumanis, 2005; Ntoumanis and Standage, 2009). Therefore, the student who has satisfied his/her basic psychological needs (i.e., autonomy, competence, and relatedness) will have a motivation to study and to engage in physical activity that is more autonomous, more self-determined, and not subjected to external pressures and will perform the tasks voluntarily, experiencing the pleasure of the activity itself (academic and/or physical education).

The results partially confirm *H3*. The results showed that only the autonomous motivation for physical education positively and significantly predicted the adolescents’ MVPA, whereas academic autonomous motivation did not predict MVPA. The explained variance of MVPA was very high, and higher than previous works (Wang, 2017). Therefore, students who showed autonomous motivation for physical education will have a higher MVPA. This result was consistent with previous studies on adolescents and children, which also used an objective measure of MVPA (Aelterman et al., 2012; Sebire et al., 2013; Kalajas-Tilga et al., 2020), as well as with the review by Zhou and Wang (2019) where they indicated that enjoyment was an element that had been consistently and positively associated with MVPA. However, academic autonomous motivation was not relevant for MVPA. Autonomous motives for attending secondary school did not have an influence on MVPA. Therefore, when students participate in physical education classes for fun, challenge, or excitement related to participation, they are more physically active. In sum, there is a differential effect of autonomous motivation on MVPA depending on the type, academic or physical education.

Contrary to that expected for *H4* “the physical self-concept perceived by adolescents is positively and indirectly related to the adolescents’ MVPA through needs satisfaction and autonomous motivation (for academic and physical education),” the results showed that there was no evidence of this indirect effect. That is, there was no influence of physical self-concept on MVPA through needs satisfaction and academic autonomous motivation. This result may also be due to the existence of multiple mediators (in this case, two mediators) between physical self-concept and MVPA, and the inter-correlation between these mediators, which could lead to multicollinearity and a distortion of the results. We should note that the paths from each mediator to the dependent variable (i.e., MVPA) were estimated by controlling for all other mediators (Hayes, 2018). Therefore, needs satisfaction and autonomous motivation (for academic and physical education) do not strengthen the relationship between physical self-concept and adolescents’ MVPA; that is, they are not relevant for higher physical self-concept leading to a higher MVPA.

### Practical Implications

This work aims to contribute to the advancement of knowledge in the specific field of the practice of physical activity, as no previous studies were found which considered the effect of personal variables and motivational processes on an objective measure of adolescents’ MVPA. According to the results obtained, and in line with the SDT postulates, physical education teachers should implement strategies that help to satisfy the three basic needs during their classes, which will contribute to fostering autonomous motivation for physical education in their students. Specifically, reinforcing and stimulating interest, curiosity, and enjoyment in the task, providing informative feedback to enhance student engagement and effort, providing feedback on student effectiveness and competence, such as frequent and immediate feedback on performance to reinforce achievements, and promoting cooperation and teamwork by showing care and attention to students, are relevant strategies for convincing students to engage in physical activity voluntarily and without pressure.

The results of the hypothesized model indicated that future interventions aimed at increasing adolescents’ MVPA should be designed to enhance students’ enjoyment when participating in physical education classes. In addition, the implementation of physical self-concept improvement strategies will not only increase personal development resources, but this improvement may also facilitate future physical activity (Babic et al., 2014; Cheon et al., 2019).

### Limitations and Future Research

The consideration of personal and motivational variables and the use of accelerometers to obtain an objective measurement of adolescents’ MVPA are important strengths of the study; however, it is also important to highlight its limitations. The design of the current study was cross-sectional, which does not allow for causal inferences between the variables. Longitudinal studies are thus necessary to determine the consistency of the effects between the different variables through time. The study was focused on analyzing the effects of basic need satisfaction (bright side), but it may also be of interest to analyze the influence of the frustration of these needs (dark side). Future work could also analyze the differential effects according to the type of activity performed by the participants, because accelerometers do not provide this information. The role of physical self-concept needs to be further addressed; although its role is unclear, there is much evidence which shows that physical self-perceptions are beneficial for adolescents’ physical activity participation (Babic et al., 2014). In this sense, it would also be interesting to identify mediating variables that enhance the relationship between physical self-concept and adolescents’ MVPA. Finally, we can consider that a larger sample size could lead to more consistent results from the data analysis.

## Conclusion

The results indicated that if students have a positive physical self-perception, their basic psychological needs in physical education class will be more satisfied. Moreover, when adolescents feel that their basic needs are satisfied (i.e., they are more physically competent, more connected to their peers, and perceive that physical activity depends on their own volition), they will perceive the activities proposed in the physical education class as fun and exciting. Thus, they will be more likely to participate in them and, consequently, will have a higher MVPA.

## Data Availability Statement

The raw data supporting the conclusions of this article will be made available by the authors, without undue reservation.

## Ethics Statement

The studies involving human participants were reviewed and approved by the Ethical Committee of the Miguel Hernandez University of Elche. DPS.JMM.01.17. Written informed consent to participate in this study was provided by the participants’ legal guardian/next of kin.

## Author Contributions

EH, JM-M, and JN: conceptualization, writing-review and editing, and supervision. JL: methodology. JL and AV-V: software. JM-M and AV-V: formal analysis. EH and LC: investigation. JN: resources. EH and AV-V: data curation. JM-M: visualization. All authors contributed to the article and approved the submitted version.

## Conflict of Interest

The authors declare that the research was conducted in the absence of any commercial or financial relationships that could be construed as a potential conflict of interest.

## Publisher’s Note

All claims expressed in this article are solely those of the authors and do not necessarily represent those of their affiliated organizations, or those of the publisher, the editors and the reviewers. Any product that may be evaluated in this article, or claim that may be made by its manufacturer, is not guaranteed or endorsed by the publisher.
